# Supplementary material for: Description and analysis of representative COVID-19 cases–A retrospective cohort study
Source: PLoS One. 2021 Jul 30;16(7):e0255513. doi: 10.1371/journal.pone.0255513 (PMC8323911; doi:10.1371/journal.pone.0255513)
Supplement: S1 Table — To assess the representativeness of our study population in comparison with the overall population infected with SARS-CoV-2, we compared the age groups sorted by age groups using the Chi-Square test. (PDF) [file pone.0255513.s001.pdf]

S1 Table: Data comparison of study population vs. overall infected population

	Age groups	n overall	% of overall population	n study population	% of study population	p-value	significance
**1**	0	9	0.7	0	0	31	*
**2**	10	25	1.9	9	1	126	not signifcantly different
**3**	20	155	11.9	99	11	0.59	not signifcantly different
**4**	30	217	16.6	147	16.4	0.9276	not signifcantly different
**5**	40	201	15.4	156	17.4	0.2358	not signifcantly different
**6**	50	244	18.7	177	19.7	0.5811	not signifcantly different
**7**	60	215	16.5	175	19.5	0.07577	not signifcantly different
**8**	70	92	7	68	7.6	0.6979	not signifcantly different
**9**	80	109	8.4	52	5.8	0.02926	*
**10**	90	27	2.1	10	1.1	0.1229	not signifcantly different
**11**	100	11	0.8	2	0.2	0.1135	not signifcantly different
**12**	missing	0	0	2	0.2	0.3238	not signifcantly different

To assess the representativeness of our study population in comparison with the overall population infected with SARS-CoV-2, we compared the age groups sorted by age groups using the Chi-Square test.
