# Supplementary material for: Description and analysis of representative COVID-19 cases–A retrospective cohort study
Source: PLoS One. 2021 Jul 30;16(7):e0255513. doi: 10.1371/journal.pone.0255513 (PMC8323911; doi:10.1371/journal.pone.0255513)
Supplement: S2 Table — Participants were asked to specify symptoms they suffered in the initial phase of the infection and symptoms which developed during the course of disease. n = 897. (PDF) [file pone.0255513.s002.pdf]

S2 Table: Initial and subsequent symptoms

	All patients	Asymptomatic patients	Mild (symptomatic outpatients)	Moderate (hospitalized)	Critical (ventilation)	Deceased
n (%)	897 (100)	54 (6.0)	713 (79.5)	97 (10.8)	16 (1.8)	17 (1.9)
Initial symptoms n (%)		Asymptomatic				
1. Fever	347 (38.7)		264 (37.0)	60 (61.9)	11 (68.8)	12 (70.6)
2. Cough	375 (41.8)		301 (42.2)	54 (55.7)	7 (43.8)	13 (76.5)
3. Sputum	38 (4.2)		30 (4.2)	6 (6.2)	1 (6.3)	1 (5.9)
4. Sore throat	229 (25.5)		203 (28.5)	23 (23.7)	3 (18.8)	1 (5.9)
5. Dyspnea	81 (9.0)		44 (6.2)	26 (26.8)	4 (25.0)	7 (41.2)
6. Muscle pain	189 (21.1)		163 (22.9)	19 (19.6)	5 (31.3)	2 (11.8)
7. Limb pain	316 (35.2)		270 (38.9)	37 (38.1)	7 (43.8)	2 (11.8)
8. Fatigue	402 (44.8)		357 (50.1)	48 (49.5)	9 (56.3)	10 (58.8)
9. Headache	368 (41.0)		323 (45.3)	38 (39.2)	5 (31.3)	2 (11.8)
10. Runny nose	173 (19.3)		160 (22.4)	11 (11.3)	2 (12.5)	0 (0.0)
11. Chest pain	80 (8.9)		68 (9.5)	9 (9.3)	1 (6.3)	2 (11.8)
12. Diarrhea	113 (12.6)		93 (13.0)	13 (13.4)	3 (18.8)	4 (23.5)
13. Nausea	52 (5.8)		39 (5.5)	9 (9.3)	2 (12.5)	2 (11.8)
14. Change in taste	239 (26.6)		209 (29.3)	26 (26.8)	3 (18.8)	1 (5.9)
15. Other	166 (18.5)		132 (18.5)	12 (12.4)	3 (18.8)	0 (0.0)
Developed symptoms n (%)		Asymptomatic				
1. Fever	134 (14.9)		110 (15.4)	22 (22.7)	2 (12.5)	0 (0.0)
2. Cough	177 (19.7)		156 (21.9)	18 (18.6)	2 (12.5)	1 (5.9)
3. Sputum	41 (4.6)		35 (4.9)	4 (4.1)	2 (12.5)	0 (0.0)
4. Sore throat	77 (8.6)		66 (9.3)	9 (9.3)	2 (12.5)	0 (0.0)
5. Dyspnea	100 (11.1)		69 (9.7)	24 (24.7)	6 (37.5)	1 (5.9)
6. Muscle pain	90 (10.0)		79 (11.1)	8 (8.3)	3 (18.8)	0 (0.0)
7. Limb pain	116 (12.9)		103 (14.5)	10 (10.3)	3 (18.8)	0 (0.0)
8. Fatigue	184 (20.5)		167 (23.4)	16 (16.5)	0 (0.0)	1 (5.9)
9. Headache	145 (16.2)		127 (17.8)	15 (15.5)	3 (18.8)	0 (0.0)
10. Runny nose	97 (10.8)		93 (13.0)	4 (4.1)	0 (0.0)	0 (0.0)
11. Chest pain	74 (8.2)		67 (9.4)	6 (6.2)	1 (6.3)	0 (0.0)
12. Diarrhea	99 (11.0)		80 (11.2)	16 (16.5)	2 (12.5)	1 (5.9)
13. Nausea	44 (4.9)		38 (5.3)	5 (5.2)	1 (6.3)	0 (0.0)
14. Change in taste	298 (33.2)		271 (38.0)	24 (24.7)	3 (18.8)	0 (0.0)
15. Other	138 (15.4)		127 (17.8)	8 (8.3)	1 (6.3)	2 (11.8)
