# Supplementary material for: Description and analysis of representative COVID-19 cases–A retrospective cohort study
Source: PLoS One. 2021 Jul 30;16(7):e0255513. doi: 10.1371/journal.pone.0255513 (PMC8323911; doi:10.1371/journal.pone.0255513)
Supplement: S3 Table — Multivariable Cox proportional hazard model analyzing age, sex, comorbidities and smoking history (in pack years). n = 897. (PDF) [file pone.0255513.s003.pdf]

S3 Table: Cox regression, Influence of variables on time to hospitalization

Variables	HR	95% Cis	p-value
Age	1.05	1.03-1.06	<0.0001
Sex<br>Female	0.59	0.41-0.85	0.005
Lung disease incl.<br>- Any lung disease<br>- Chronic Asthma with medication<br>- Any lung surgery	1.09	0.72-1.64	0.688
Smoking history (py)	1.00	0.99-1.02	0.801
Coronary heart disease	1.45	0.70-3.00	0.313
Hypertension	1.09	0.70-1.68	0.702
Diabetes mellitus (Type 1 and 2)	1.39	0.77-2.50	0.273

Multivariable Cox proportional hazard model analyzing age, sex, comorbidities and smoking history (in pack years). n=897
