# Supplementary figures and images for: Description and analysis of representative COVID-19 cases–A retrospective cohort study
Source: PLoS One. 2021 Jul 30;16(7):e0255513. doi: 10.1371/journal.pone.0255513 (PMC8323911; doi:10.1371/journal.pone.0255513)

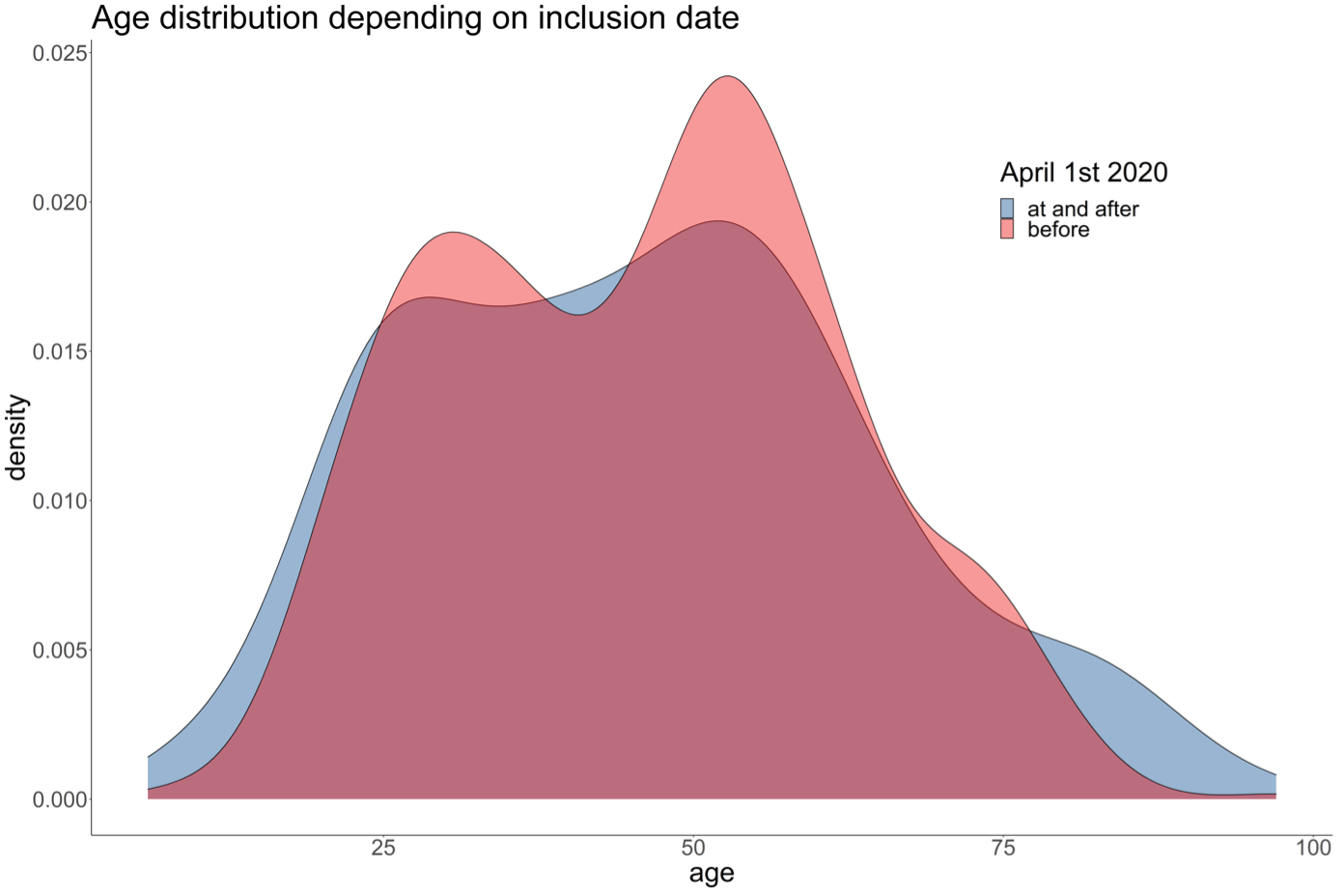

Supplement: S1 Fig — In the early part of the epidemic many cases returned from skiing holidays. We display the age distribution before and after the border closure considering 14 days of an incubation period (i.e. April 1st, 2020). n = 897. (PNG) [file pone.0255513.s004.png]
