# Supplementary material for: Description and analysis of representative COVID-19 cases–A retrospective cohort study
Source: PLoS One. 2021 Jul 30;16(7):e0255513. doi: 10.1371/journal.pone.0255513 (PMC8323911; doi:10.1371/journal.pone.0255513)
Supplement: S1 File — Copy of the questionnaire distributed to consenting participants. (PDF) [file pone.0255513.s005.pdf]

UNIVERSITÄTS
KLINIKUM
HEIDELBERG

COVID-19 Fragebogen

Bitte beantworten Sie die unten stehenden Fragen.

Die Umfrage wird voraussichtlich 10-15 Minuten dauern. Die Umfrage dient dazu, den Diagnoseprozess und die Erkrankung und damit verbunde Faktoren Ihrer SARS-CoV-2 (neuartiges Coronavirus) Infektion zu verstehen. Mit Ihren Antworten helfen Sie medizinischen Einrichtungen, entsprechende Maßnahmen zu planen, sodass Patienten eine bessere medizinische Versorgung erhalten können.
Jede Frage kann übersprungen oder ausgelassen werden.

Allgemeine Fragen

Wie lautet Ihre Studien-ID?<br>*Sie finden die Studien-ID auf dem Anschreiben links unten. (z. B. HD-S-586)*	______________________
Wie lautet die Postleitzahl Ihres Wohnortes ?	______________________
Wie lautet Ihr Geburtsmonat?	______________________<br>(Bitte geben Sie eine Zahl zwischen 1 und 12 an)
Wie lautet Ihr Geburtsjahr ?	______________________<br>(Bitte schreiben Sie Ihr Geburtsjahr im Format 1950)
Ihr Geschlecht ?	◯ Männlich<br>◯ Weiblich<br>◯ Divers
Wie groß sind Sie ? (in cm)	______________________
Wieviel Kilogramm wiegen Sie ?	______________________
Wie ist Ihre Wohnsituation ?	◯ Allein<br>◯ in Partnerschaft<br>◯ Sonstiges
Anzahl Personen im Haushalt ?	______________________
Davon Anzahl Personen unter 18 Jahren ?	______________________

Hatten Sie während Ihrer COVID-19 Infektion Symptome?	◯ Ja<br>◯ Nein
Falls ja: Frühestes Auftreten der Symptome während Ihrer COVID-19 Infektion ?	____________________<br>(TT-MM-JJJJ)
Falls ja: Erste Symptome, seit Sie mit COVID-19 infiziert waren (mehrere Möglich) ?	☐ Erhöhte Temperatur / Fieber<br>☐ Husten<br>☐ Auswurf<br>☐ Halsschmerzen<br>☐ Blutiges Abhusten<br>☐ Atemnot<br>☐ Muskelschmerzen<br>☐ Gliederschmerzen<br>☐ Müdigkeit<br>☐ Kopfschmerzen<br>☐ Laufende Nase<br>☐ Schmerzen in der Brust<br>☐ Durchfall<br>☐ Übelkeit / Erbrechen<br>☐ Veränderung des Geschmacks<br>☐ Sonstiges
Bei erhöhter Temeratur/Fieber: Was war Ihre höchste Körpertemperatur seit Ihrer Infektion ?	____________________
Wenn Sonstiges, was ?	____________________
Haben sich Ihre Symptome im Verlauf der Erkrankung verschlechtert ?	◯ Ja<br>◯ Nein
Falls ja: An welchem Datum haben sich Ihre Symptome verschlechtert ?	____________________<br>(TT-MM-JJJJ)
Haben sich Ihre Symptome im Verlauf verändert ?	◯ Ja<br>◯ Nein
Falls ja: Welche Symptomatik ist neu hinzugekommen ?	☐ Erhöhte Temperatur / Fieber<br>☐ Husten<br>☐ Auswurf<br>☐ Halsschmerzen<br>☐ Blutiges Abhusten<br>☐ Atemnot<br>☐ Muskelschmerzen<br>☐ Gliederschmerzen<br>☐ Müdigkeit<br>☐ Kopfschmerzen<br>☐ Laufende Nase<br>☐ Schmerzen in der Brust<br>☐ Durchfall<br>☐ Übelkeit / Erbrechen<br>☐ Veränderung des Geschmacks<br>☐ Sonstiges

Bei erhöhter Temeratur/Fieber: Was war Ihre höchste Körpertemperatur?	____________
Wenn Sonstiges, was ?	____________
An welchem Datum wurde Ihre COVID-19 Diagnose bestätigt (positives Testergebnis) ?	____________ (TT-MM-JJJJ)
Wo wurden Sie getestet ?	◯ Universitätsklinikum<br>◯ Drive-In Klinik<br>◯ Externe Testklinik<br>◯ Mobile Teststation<br>◯ Praxis
Falls "Externe Testklinik": In welcher externen Testklinik ?	____________
War ein Test vorab negativ ?	◯ Ja<br>◯ Nein
Falls ja: An welchem Datum war ein Test vorab negativ ?	____________ (TT-MM-JJJJ)
Ist Isolierung bereits aus eigener Initiative erfolgt ?	◯ Ja<br>◯ Nein
Falls ja: Ab wann ?	____________ (TT-MM-JJJJ)
Geschätzte Anzahl der Kontaktpersonen von Beginn der Symptome bis zur Isolation ? (inklusive Personen im Haushalt)	____________
Wo waren Sie in Isolation, nachdem die Diagnose gestellt wurde ?	☐ Zuhause<br>☐ Krankenhaus<br>☐ Anderer Ort
Falls "Anderer Ort": Welcher ?	____________
Wo vermuten Sie, sich mit COVID-19 infiziert zu haben ?	◯ Weiß nicht / nicht sicher<br>◯ Sozialkontakt<br>◯ Arbeitskontakt<br>◯ Kontakt in Universität / Schule / Kindergarten von Ihnen oder einem Kind in der Familie<br>◯ Reise in Risikogebiet<br>◯ Sonstige
Falls "Reise in Risikogebiet": Welches Risikogebiet ?	____________
Falls "Sonstige": Bitte beschreiben:	____________

Wie krank waren Sie, als die Diagnose gestellt wurde ?	◯ Normale uneingeschränkte Aktivität wie vor der Erkrankung.<br>◯ Einschränkung bei körperlicher Anstrengung, aber gehfähig; leichte körperliche Arbeit bzw. Arbeit im Sitzen (z.B. leichte Hausarbeit oder Büroarbeit) möglich.<br>◯ Gehfähig, Selbstversorgung möglich, aber nicht arbeitsfähig; kann mehr als 50% der Wachzeit aufstehen.<br>◯ Nur begrenzte Selbstversorgung möglich; 50% oder mehr der Wachzeit an Bett oder Stuhl gebunden.<br>◯ Völlig pflegebedürftig, keinerlei Selbstversorgung möglich; völlig an Bett oder Stuhl gebunden.
Waren Sie stationär im Krankenhaus ?	◯ Ja<br>◯ Nein
Falls ja: Ab wann waren Sie stationär im Krankenhaus ? (Datum)	______________________<br>(TT-MM-JJJJ)
Falls ja: Für wie viele Tage waren Sie stationär im Krankenhaus ?	______________________
Falls ja: Waren Sie auf der Intensivstation ?	◯ Ja<br>◯ Nein
Falls ja: Mussten Sie künstlich beatmet werden ?	◯ Ja<br>◯ Nein
Falls Sie Symptome hatten:<br>Wann sind Ihre Symptome verschwunden ? (Datum)	______________________<br>(TT-MM-JJJJ)
Wurde zum Ende der Quarantäne ein Kontrollabstrich vorgenommen, der **negativ** war ?	◯ Ja<br>◯ Nein
Falls ja: Wann wurde der negative Kontrollabstrich zum Ende der Quarantäne vorgenommen ? (Datum)	______________________<br>(TT-MM-JJJJ)
Wurde zum Ende der Quarantäne ein Kontrollabstrich vorgenommen, der **positiv** war ?	◯ Ja<br>◯ Nein
Falls ja: Wann wurde der positive Kontrollabstrich zum Ende der Quarantäne vorgenommen ? (Datum)<br>*Bei mehreren positiven Kontrollabstrichen: Datum des letzten positiven Abstrichs*	______________________<br>(TT-MM-JJJJ)
Wo sind Sie jetzt ?	◯ Aus der Isolation entlassen<br>◯ Noch im Krankenhaus
Heutiges Datum / Datum der Befragung	______________________<br>(TT-MM-JJJJ)

Welche der folgenden Erkrankung(en) der Lunge haben Sie ? (mehrere möglich)	☐ Asthma bronchiale<br>☐ Chronisch obstruktive Lungenerkrankung (COPD)<br>☐ Atemstörungen im Schlaf<br>☐ Obstruktives Schlafapnoesyndrom (OSAS)<br>☐ Interstitielle Lungenerkrankung (Erkrankungen des Lungengerüsts)<br>☐ Lungenkrebs<br>☐ Andere<br>☐ Keine
Falls "Andere":<br>Welche andere/n Erkrankung/en der Lunge haben Sie ?	______________________________
Sind Sie schon einmal an der Lunge operiert worden?	◯ Ja<br>◯ Nein
Haben Sie eine Erkrankung/en des Herz-/Kreislaufsystems ? (z. B. Bluthochdruck oder Gefäßverkalkung)	◯ Ja<br>◯ Nein
Falls ja: Welche Erkrankung/en des Herz-/Kreislaufsystems haben Sie ?	☐ Erkrankung der Herzkranzgefäße<br>☐ Herzrhythmusstörungen<br>☐ Herzversagen/Herzinsuffizienz<br>☐ Schlaganfall<br>☐ Bluthochdruck (Hypertonie)<br>☐ Periphere arterielle Verschlusskrankheit (arterielle Durchblutungsstörung der Beine)<br>☐ Andere
Haben Sie eine Erkrankung/en der Niere ?	◯ Ja<br>◯ Nein
Falls ja: Welche Erkrangkung/en der Niere haben Sie ?	◯ Chronisches Nierenversagen mit Dialyse<br>◯ Chronisches Nierenversagen ohne Dialyse<br>◯ Erfolgte Nierentransplantation<br>◯ Andere
Haben Sie eine Erkrankung/en der Leber?	◯ Ja<br>◯ Nein
Falls ja: Welche Erkrankung/en der Leber haben Sie ?	◯ Fettleber (Nicht-alkoholische Fettleber, NASH)<br>◯ Zirrhose<br>◯ Erfolgte Lebertransplantation<br>◯ Andere

Weitere Erkrankungen

	Ja	Nein
Haben Sie Diabetes mellitus?	◯	◯
Haben Sie eine Autoimmunerkrankung?	◯	◯
Sind Sie mit HIV infiziert?	◯	◯
Haben oder hatten Sie eine Krebserkrankung (außer Lungenkrebs)?	◯	◯
Haben Sie weitere Erkrankung/en?	◯	◯

Falls Sie Diabetes mellitus haben:
Spritzen Sie regelmäßig Insulin ?

◯ Ja
◯ Nein

Falls Sie eine Autoimmunerkrankung haben:
Welche Autoimmunerkrankung haben Sie?

☐ Rheumatoide Arthritis (Rheuma)
☐ Multiple Sklerose
☐ Andere

Falls "Andere":
Welche andere Autoimmunerkrankung/en haben Sie ?

____

Falls Sie weitere Erkankung/en haben:
Welche weitere/n Erkrankung/en haben Sie ?

____
(Bei mehreren Erkankungen bitte eine neue Zeile verwenden)

Haben Sie vor dem Beginn der Symptome regelmäßig Medikamente eingenommen ?

◯ Ja
◯ Nein

Falls ja: Welche Medikamente haben Sie vor dem Beginn der Symptome regelmäßig eingenommen ?

Bitte geben Sie ein Medikamenten-/Präparatname pro Zeile an und geben Sie an, wann/wie oft Sie das Medikament nehmen im Stil morgens-mittags-abends-nachts.

Eine 0 steht für "Medikament zu dieser Zeit nicht genommen",
eine 1 steht dafür für "Medikament in der angegebenen Dosis zu dieser Zeit genommen",
eine 2 steht für "Medikament in zweifacher Dosis zu dieser Zeit genommen".

Z. B. Nur morgens zwei Tabletten Pantoprazol (20mg) würde also als
"Pantoprazol 20mg 2-0-0-0"
geschrieben werden,
Mittags und nachts eine Tablette ASS (100mg) als
"ASS 100mg 0-1-0-1"

Sind Sie schwanger ?

◯ Ja
◯ Nein

**Nehmen Sie eines der folgenden Medikamente?**	Ja	Nein	Weiß nicht
Nehmen Sie Medikamente ein mit einem Wirkstoff, der auf "-pril" endet? (z.B. Benazepril, Captopril, Cilazapril, Enalapril, Fosinopril, Imidapril, Lisinopril, Moexipril)	○	○	○
Nehmen Sie Medikamente ein mit einem Wirkstoff, der auf "-sartan" endet? (z. B. Azilsartan (Prodrug: Azilsartanmedoxomil), Candesartan, Eprosartan, Fimasartan, Irbesartan, Losartan, Milfasartan, Olmesartan, Pomisartan, Pratosartan, Ripisartan, Saprisartan, Tasosartan, Telmisartan, Valsartan)	○	○	○
Nehmen Sie Medikamente ein, die das Immunsystem schwächen? (z.B. "Cortison" oder Medikamente nach einer Transplantation)	○	○	○
Nehmen Sie Medikamente gegen eine Autoimmunerkrankung ein?	○	○	○
Erhalten Sie aktuell oder im vergangenen Monat eine Chemotherapie?	○	○	○
Nehmen Sie regelmäßig antientzündliche Schmerzmittel (z.B. Ibuprofen, Paracetamol etc.) ein	○	○	○

Falls Sie regelmäßig antientzündliche Schmerzmittel nehmen: Seit wann nehmen Sie regelmäßig antientzündliche Schmerzmittel ein ? ____________________

Rauchen Sie ?

- ○ Ja
- ○ Nein; nicht mehr
- ○ Nein; niemals

Bei "Ja":
Seit wie vielen Jahren rauchen Sie ? ____________________

Bei "Ja": Wie viele Zigaretten rauchen Sie durchschnittlich am Tag ? ____________________

Bei "Nein; nicht mehr":
Für wie viele Jahre haben Sie in etwa geraucht ? ____________________

Bei "Nein; nicht mehr": Wie viele Zigaretten haben Sie in diesem Zeitraum in etwa durchschnittlich pro Tag geraucht ? ____________________

Wie of fühlten Sie sich im Verlauf der LETZTEN 2 WOCHEN durch die folgenden Beschwerden beeinträchtigt ?

	Überhaupt nicht	An einzelnen Tagen	An mehr als der Hälfte der Tage	Beinahe jeden Tag
Wenig Interesse oder Freude an Ihren Tätigkeiten	○	○	○	○
Niedergeschlagenheit, Schwermut oder Hoffnungslosigkeit	○	○	○	○

Wie war Ihre allgemeine Stimmung während Ihrer Isolation ?	◯ Schlecht<br>◯ Eher Schlecht<br>◯ Mittelmäßig<br>◯ Eher Gut<br>◯ Gut
Bevor COVID-19 bei Ihnen diagnostiziert wurde, wie haben Sie das Risiko einer Ansteckung mit COVID-19 für sich eingeschätzt ?	◯ Hoch<br>◯ Moderat/Mittel<br>◯ Minimal<br>◯ Nicht vorhanden
Wie ist Ihre berufliche Situation ?	☐ Selbstständig<br>☐ Angestellt (Vollzeit)<br>☐ Angestellt (Teilzeit)<br>☐ Im Ruhestand<br>☐ Arbeitslos<br>☐ Schüler<br>☐ Student<br>☐ in Ausbildung<br>☐ andere
Was sind die Auswirkungen von COVID-19 auf Ihre Arbeit ?	☐ Arbeit wie gewohnt<br>☐ Remote-Office-Arbeit (Home-Office)<br>☐ Krankenstand<br>☐ Kurzarbeit<br>☐ Urlaub<br>☐ Arbeit geschlossen während Ausbruch<br>☐ Entlassung<br>☐ Im Ruhestand / Keine Arbeit<br>☐ Keine Aufträge/Kunden (Selbstständig)<br>☐ Wiedereinstieg in Berufstätigkeit
Welche Informationsquelle/n nutzen sie, wenn Sie sich über Infektionskrankheiten wie COVID-19 informieren ?	☐ Twitter<br>☐ Facebook<br>☐ Wikipedia<br>☐ Robert-Koch-Institut<br>☐ Radio<br>☐ Fernsehen / TV<br>☐ Nachrichtenportale<br>☐ Zeitungen<br>☐ Bundesgesundheitsministerium<br>☐ medizinische (online) Fachliteratur<br>☐ andere Internetquellen<br>☐ Sonstige
Bei "Sonstige": Welche sonstige Informationsquelle/n nutzen sie, wenn Sie sich über Infektionskrankheiten wie COVID-19 informieren ?	____________________

Welcher/n Informationsquelle/n vertrauen Sie NICHT, wenn Sie sich über Infektionskrankheiten wie COVID-19 informieren ?	☐ Twitter<br>☐ Facebook<br>☐ Wikipedia<br>☐ Robert-Koch-Institut<br>☐ Radio<br>☐ Fernsehen / TV<br>☐ Nachrichtenportale<br>☐ Zeitungen<br>☐ Bundesgesundheitsministerium<br>☐ medizinische (online) Fachliteratur<br>☐ andere Internetquellen<br>☐ Sonstige
Bei "Sonstige": Welcher/n sonstige/n Informationsquelle/n trauen Sie nicht, wenn Sie sich über Infektionskrankheiten wie COVID-19 informieren ?	________________________

Wieviel hat Sie Ihre COVID-19 Erkrankung bisher gekostet (Schätzung, freiwillige Angaben)

Einkommensverlust (Euro)	________________________
Ausgaben Gesundheitswesen (Zuzahlung) (Euro)	________________________
Übernachtungskosten (Hotel, Transportkosten etc.) (Euro)	________________________
Sonstige Kosten (Euro)	________________________

Vielen Dank für Ihre Zeit und Ihre Mithilfe.
Gibt es noch etwas, das Sie uns Mitteilen wollen ? Gerne können Sie Feedback sonstige Kommentare verwenden auf dieser Seite vermerken.
Bitte beachten Sie, dass wir Ihnen auf diese Kommentare nicht antworten können. Bei Rückfragen können Sie uns telefonisch erreichen unter: 06221 522-1890 oder per E-Mail unter:
covidsurvey@rhein-neckar-kreis.de

Description of variables assessed in questionnaire

Variable	Type	Definition
Birth month	Continuous	01-12 (MM)
Birth year	Continuous	4-digit number (YYYY)
Sex	Binary/Categorical	Male, female, other
Height	Continuous	In centimeter (cm)
Weight	Continuous	In kilogram (kg)
Housing Situation	Categorical	Alone, in relationship with partner, other
Number of children <18 at home	Continuous	Children living in same household
Presumed source of infection	Categorical	Not sure, social encounter, Work, Encounter in school, university, kindergarten, travel, other

Measured Symptom	Type	Definition
Any symptoms<br>If 1 (yes), then:	Binary	0: None, 1: Yes
Fever	Continuous	Peak temperature (°C)
Cough	Binary	0: None, 1: Yes
Cough with sputum	Binary	0: None, 1: Yes
Cough with bloody sputum	Binary	0: None, 1: Yes
Sore throat	Binary	0: None, 1: Yes
Dyspnea	Binary	0: None, 1: Yes
Myalgia	Binary	0: None, 1: Yes
Fatigue	Binary	0: None, 1: Yes
Headache	Binary	0: None, 1: Yes
Rhinorrhea	Binary	0: None, 1: Yes
Chest pain	Binary	0: None, 1: Yes
Diarrhea	Binary	0: None, 1: Yes
Nausea/Emesis	Binary	0: None, 1: Yes
Dysgeusia	Binary	0: None, 1: Yes
Other	Qualitative	Free text answer

Description of variables assessed in questionnaire

Treatment	Type	Definition
Home isolation	Binary	0: No, 1: Yes
Hospitalization	Binary	0: No, 1: Yes (Admission to hospital)
Day of admission	Continuous	Date (DD/MM/YYYY)
Duration of hospital stay	Continuous	Days
ICU	Binary	0: No, 1: Yes (Admission to intensive care unit)
Mechanical ventilation or ECMO	Binary	0: No, 1: Yes (Requiring mechanical ventilation)

**Risk Factor**	**Type**	**Definition**
COPD	Binary	0: No, 1: Yes
Bronchial asthma	Binary	0: No, 1: Yes
Interstitial Lung Disease	Binary	0: No, 1: Yes
Nocturnal dyspnea	Binary	0: No, 1: Yes
Lung cancer	Binary	0: No, 1: Yes
Malign cancer (excluding lung)	Binary	0: No, 1: Yes
Cardiovascular disease	Binary	0: No, 1: Yes
Breakdown	Categorical	Coronary heart disease, Arrythmia, Heart failure, stroke, hypertension, peripheral arterial occlusive disease, other
Kidney disease	Binary	0: No, 1: Yes
Breakdown	Categorical	If yes, kidney failure with/without dialysis, kidney transplant, other

Description of variables assessed in questionnaire

Liver disease	Binary	0: No, 1: Yes
Breakdown	Categorical	If yes, hepatic steatosis, cirrhosis, liver transplant, other
Autoimmune-Disease	Binary	0: No, 1: Yes
Breakdown	Categorical	If yes, rheumatoid arthritis, multiple sclerosis, other
Diabetes	Binary	0: No, 1: Yes
HIV	Binary	0: No, 1: Yes
Obesity	Continuous	Body mass index >30 kg/m^2
Other	Qualitative	Free text answer
Medication	Qualitative	Free text answer (Name, daily dosis)
ACE-Inhibitors	Binary	0: No, 1: Yes
Sartans	Binary	0: No, 1: Yes
Immunsuppressives	Binary	0: No, 1: Yes
Chemotherapy	Binary	0: No, 1: Yes
Drugs against autoimmune diseases	Binary	0: No, 1: Yes
NSAID	Binary	0: No, 1: Yes
Smoking	Categorical	Yes, former, never
Pack Years	Continuous	(Cigarettes per day/Pack size) * Years
Pregnancy	Binary	0: No, 1: Yes
