# Supplementary material for: Description and analysis of representative COVID-19 cases–A retrospective cohort study
Source: PLoS One. 2021 Jul 30;16(7):e0255513. doi: 10.1371/journal.pone.0255513 (PMC8323911; doi:10.1371/journal.pone.0255513)
Supplement: S1 Text — Description of variables. (PDF) [file pone.0255513.s006.pdf]

S1 Text. Additional methods

In the survey gender was assessed in four categories (male, female, other, missing).

We calculated the participants age based on the year of birth. The presence of children < 18 years variable is a dichotomized version of a survey question that assessed the number of children younger than 18 in the household. Any number of children was considered presence of children for our analysis.

We calculated the pack years from data on the duration participants were smokers and the amount smoked of cigarettes per day.

The variable for immune suppression was dichotomized from the presence of immune suppressants in the survey answers.

We calculated the times from symptom onset to diagnosis and hospitalization based on the given dates for each. Therefore, where no date was available (e.g. when asymptomatic patients never had a symptom onset) no times could be calculated.

The symptom composition with disease in the descriptive analysis were defined as symptoms at diagnosis and in the course of disease. Symptoms at diagnosis and during the course of disease are reported separately in the supplement.

All other variables and those in the descriptive analysis were directly taken from the survey data without further modifications.
