# Supplementary material for: Description and analysis of representative COVID-19 cases–A retrospective cohort study
Source: PLoS One. 2021 Jul 30;16(7):e0255513. doi: 10.1371/journal.pone.0255513 (PMC8323911; doi:10.1371/journal.pone.0255513)
Supplement: Attachment — Submitted filename: Response to reviewers.docx [file pone.0255513.s007.docx]

| **Journal Comments to the Author** | **Response from Author** |
| --- | --- |
| 1. Please ensure that your manuscript meets PLOS ONE's style requirements, including those for file naming. The PLOS ONE style templates can be found at | Thank you for this comment. We have made appropriate adaptions throughout the document. |
| 2. Thank you for stating the following in the Funding Section of your manuscript:  [This work was supportedby internal COVID funds of the Heidelberg University Hospital]  We note that you have provided funding information that is not currently declared in your Funding Statement. However, funding information should not appear in the Acknowledgments section or other areas of your manuscript. We will only publish funding information present in the Funding Statement section of the online submission form.  Please remove any funding-related text from the manuscript and let us know how you would like to update your Funding Statement. Currently, your Funding Statement reads as follows:   [The authors received no specific funding for this work.] | We excluded the funding statement in the manuscript and wish to keep the following statement  (“This work was supported by internal COVID funds of the Heidelberg University Hospital.”) |
| 3. Please include captions for your Supporting Information files at the end of your manuscript, and update any in-text citations to match accordingly. Please see our Supporting Information guidelines for more information: [http://journals.plos.org/plosone/s/supporting-information](about:blank). | Thank you for this remark. Appropriate changes have been incorporated throughout. |

| **Reviewer 1 Comments to the Author** | **Response from Author** |
| --- | --- |
| The authors conducted a retrospective survey on the COVID-19 patients in Southern German, described clinical features and assessed potential risk factors for hospitalization. Overally, the article is well written and well designed. In my opinion, it need only some minor revisions. | We appreciate the reviewer’s comment. |
| 1. Statistics: what kind of multivariate logistic regression model was performed, backward stepwise or others？ | Thank you for this comment! We did not use any stepwise model selection algorithms but selected the variables for the multivariate analysis from a series of univariate screening models.  We clarified this in lines 134-138 |
| 2. I think hospitalization is influenced potentially by many factors, e.g., disease severity, compliance of patients, the affordability of health care system, and even management policy in each country. For example, in China, even asymptomatic and mild COVID-19 patients are hospitalized. So, I suggest this should be discussed in section of limitation. | We appreciate the reviewer’s comment and have added this to the limitation section (line 443-446) |
| 3. P value is not a trend. So the sentences should be expressed in a more appropriate way (Page 20, line 358, line 364) | Thank you for this comment, we have changed the sentences (line 417-423) |

| **Reviewer 3 Comments to the Author** | **Response from Author** |
| --- | --- |
| 1. Patients enrolled from February 7 to June 30 were included in the follow-up, and the paper needs to explain when the retrospective investigation was conducted. | Thank you for your remark. We added information on the period of conduct (line 60-61). Data collection started on March 19, 2020 and concluded on June 30, 2020. |
| 2. There are major problems with the study design. The study design of this paper is not a retrospective cohort study, not based on the exposure of certain risk factors, but divided the outcomes of the cases into different study levels and collected relevant data retrospectively, which should be a cross-section investigation design and then do case-control study and risk factors analysis. | We appreciate the reviewer’s valuable feedback. A case control study or prospective cohort study might have been of higher value. However, given the dynamics of the early pandemic this was not feasible.  We believe that or study fills all the formal criteria for a retrospective cohort study in that it:   - Describes a cohort selected by the occurrence of an outcome - Retrospectively evaluates risk factors that might have contributed to said diseases, i.e. accumulated significantly in our population   Retrospectively assesses said risk factors contribution to the severity of the disease (thus stratifying the populations into groups, e.g. age, smoking status and using this stratification as case-control setup) |
| 3. In the study, 30% cases lost to follow-up may affect the distribution of the pathogen of the disease, making the results less reliable. So, suggest to analysis the basic situation of the lost. | Thank you for this comment. We highlighted this limitation of the study at the end of our discussion. (line 454-455) In addition, we compared the age structure of our study population to the overall population in the district tested positive for SARS-CoV-2 to identify potential underrepresentation of age groups. (line 129-131; line 194-197) |
| 4. Regarding to the risk factor analysis, it is more appropriate for the authors to conduct univariate analysis firstly to find meaningful variate and then do multiple logistic regression. | We appreciate the reviewer’s comment and have revised the appropriate methods section to indicate that we had done a univariate analysis first. The section should be clearer now. (Line 139-143) |
| 5. The authors should notice that all COVID-19 including are required to hospital for isolation in China. | We are thankful for this information and have acknowledged this. This is not the case in Germany. |
| 6. Line 212-213, page 19, please check the number of percent and its corresponding type of patients. It is inconsistent with the figure presented in Table 2 | We appreciate that the reviewer pointed this out. We have identified the mistake and included appropriate changes but could not find inconsistencies on page 19. |

| **Reviewer 4 Comments to the Author** | **Response from Author** |
| --- | --- |
| Discussion  Data regarding BMI index is missing in the study and it has also been related to worse outcomes in other large community-based cohort study (Gao M, Piernas C, Astbury NM, Hippisley-Cox J, O'Rahilly S, Aveyard P, Jebb SA. Associations between body-mass index and COVID-19 severity in 6•9 million people in England: a prospective, community-based, cohort study. Lancet Diabetes Endocrinol. 2021 Apr 28:S2213-8587(21)00089-9). Please, add this comment to the discussion section. | Thank you for this helpful comment! Unfortunately, the data collected on weight in our study was not reliable, which led us to exclude the information. However, we added this risk factor to our discussion (line 417-424). |
| Supplementary material  Please translate the questionnaire into English | We appreciate the reviewer’s feedback and have added supplement tables that depict the assessed variables of the questionnaire. |
