# Supplementary material for: Description and analysis of representative COVID-19 cases–A retrospective cohort study
Source: PLoS One. 2021 Jul 30;16(7):e0255513. doi: 10.1371/journal.pone.0255513 (PMC8323911; doi:10.1371/journal.pone.0255513)
Supplement: Attachment — Submitted filename: Response to reviewers_R2.docx [file pone.0255513.s008.docx]

| **Journal Comments to the Author** | **Response from Author** |
| --- | --- |
| 1. Please review your reference list to ensure that it is complete and correct. If you have cited papers that have been retracted, please include the rationale for doing so in the manuscript text, or remove these references and replace them with relevant current references. Any changes to the reference list should be mentioned in the rebuttal letter that accompanies your revised manuscript. If you need to cite a retracted article, indicate the article’s retracted status in the References list and also include a citation and full reference for the retraction notice. | We have checked our references and ensured only appropriate references were included. We did not include retracted publications. |

| **Reviewer 3 Comments to the Author** | **Response from Author** |
| --- | --- |
| 1. In my opinion, cohort study means classification is made according to exposure factor, generally one factor. For example, if we want to study the association between smoking (exposure factor) and lung cancer (outcome variable), the study population are classified as smoking group and non-smoking group according to their smoking history status. And then follow them, observe whether they develop lung cancer or not after a period of times. Usually, only one exposure factor is used as the classified variable. Conversely, the outcome variables may be more than one. In the above mentioned example, aside from lung cancer, the outcome variables may be hypertention, stroke or others. Here, we calculate the relative ration (RR) rather than the odds ratio (OR) to show the degree of association between the two factors (here is smoking and lung cancer). So, if the author declared that they used a cohort study, please explain what the exposure variable and outcome variable were in their study. | We appreciate the reviewer’s feedback and generally agree with the raised points and assessment. Coherent with the design of a cross-sectional study, our study was constructed to show correlations between an exposure and outcome variable. Due to the retrospective design, data for both were collected at the same time and the Odds ratio was calculated based on a logistic regression model. Nonetheless, we still are of the opinion that this study could also be described as a cohort study due to the reasons mentioned below.  In their textbook on medical statistics, Aviva and Sabin define a (retrospective) cohort study as follows: “A cohort study takes a group of individuals and usually follows them forward in time, the aim being to study whether exposure to a particular aetiological factor will affect the incidence of a disease outcome in the future. If so, the factor is generally known as a risk factor for the disease outcome. […] Although most cohort studies are prospective, historical cohorts are occasionally used: these are identified retrospectively and relevant information relating to outcomes and exposures of interest up to the present day ascertained using medical records and memory.”  (Petrie, Aviva, and Caroline Sabin. *Medical Statistics at a Glance*, John Wiley & Sons, Incorporated, 2009. *ProQuest Ebook Central*, http://ebookcentral.proquest.com/lib/ub-heidelberg/detail.action?docID=1561073. Created from ub-heidelberg on 2021-06-24 15:30:09.)  We believe that this definition of a historical or retrospective cohort applies to our study because a defined group (in this case patients who tested positive for SARS-CoV-2) was followed backwards in time to identify whether the exposure of an aetiological factor (i.e. age, sex heart disease, lung disease, diabetes, smoking history, living with children) had an effect on the defined outcome (i.e. hospitalization, severity of disease).  Instead of classifying by exposure variables (i.e. age, sex heart disease, lung disease, diabetes, smoking history, living with children) as usually done in cohort studies, we decided to classify the population by the outcome variable (hospitalization and no hospitalization) due to the relatively large number of variables considered in the analysis. This in our opinion allowed a better and more structured way of presenting our results to the reader without defying the essence of a cohort study.  Because of the complex demographic of our study population, we needed to adjust for confounding factors. To do so, we created a logistic regression model for our (binary) exposure variables and therefore, present the results as odds ratios.  Even though we, alongside other reviewers, believed to have fulfilled the criteria for a retrospective cohort study, we acknowledge that our study also meets criteria for a cross-sectional study design. If deemed necessary by the editor, we will change the title and methods to cross-sectional instead of cohort. |
